# Supplementary material for: Temporal dynamics of viral fitness and the adaptive immune response in HCV infection
Source: eLife. 2025 Aug 29;13:RP102232. doi: 10.7554/eLife.102232 (PMC12396813; doi:10.7554/eLife.102232)
Supplement: Supplementary file 1. [file elife-102232-supp1.docx]

# Supplementary information

**Supplementary File 1 – Summary of epitope selection and (IFN-γ) ELISPOT assay responses in subjects who cleared infection.**

| **Subject ID** | **Disease Outcome** | **Genotype** | **No. of positive IFN-γ ELISPOT** | **No. of epitopes underwent escape** | **Epitope ^a^** |  |
| --- | --- | --- | --- | --- | --- | --- |
| 300360 | Clearer | 3a | 3 | 0 | YLTAYQATV |  |
|  |  |  |  |  | SVIDCNVAV |  |
|  |  |  |  |  | ATDALMTGF |  |
| 300277 | Clearer | 3a | 6 | 0 | NLPGCSFSI |  |
|  |  |  |  |  | RLWHYPCTV |  |
|  |  |  |  |  | HSNIEEVAL |  |
|  |  |  |  |  | ILAGYGAGV |  |
|  |  |  |  |  | AWETARHTPV |  |
|  |  |  |  |  | WLGNIIMYA |  |
| MCRL0786FX | Clearer | 1a | 2 | 0 | GPRLGVRAT |  |
|  |  |  |  |  | ATDALMTGF |  |
| 400087 | Clearer | 1b | 3 | 0 | VHYPYRLWHY |  |
|  |  |  |  |  | GAPITYSTY |  |
|  |  |  |  |  | SFSIFLLAL |  |
| 300364 | Clearer | 1a | 2 | 0 | KSKRTPMGF |  |
|  |  |  |  |  | ATDALMTGF |  |
| 300231 | Clearer | 3a | 1 | 0 | RAQAPPPSW |  |
| 400089 | Clearer | 1b | 1 | 0 | GPRLGVRAT |  |
| 300164 | Clearer | 3a | 1 | 0 | DYPYRLWHY |  |
| **Total** |  |  | 19 | 0 |  |  |
| * - Epitope not used due to length criteria being 9-10mer | | | | | | |
| a - Epitopes with ≥ 25 SFU / million cells in IFN-γ ELISPOT assay | | | | | | |

**Supplementary File 2 – Summary of epitope selection and positive (IFN-γ) ELISPOT assay responses in subjects who developed chronic infection.**

| **Subject ID** | **Disease Outcome** | **Genotype** | **No. of potential Epitopes** | **No. of selected Epitopes** | **No. of positive IFN-γ ELISPOT** | **No. of epitopes underwent escape** | **Epitope ^a^** | |
| --- | --- | --- | --- | --- | --- | --- | --- | --- |
| 300023 | Chronic | 1a | 2213 | 45 | 8 | 2 | (K/N)SKRTPMGF | |
|  |  |  |  |  |  |  | RAEA(Q/H)LHAW | |
|  |  |  |  |  |  |  | CINGVCWTV | |
|  |  |  |  |  |  |  | VLSDFKTWL | |
|  |  |  |  |  |  |  | AEVIAPAVQT | |
|  |  |  |  |  |  |  | FAWYLKGKW | |
|  |  |  |  |  |  |  | AELIEANLLW | |
|  |  |  |  |  |  |  | WLGNIIMFA | |
| 300240 | Chronic | 3a | 1188 | 70 | 3 | 2 | RAQA(P/L)PPSW | |
|  |  |  |  |  |  |  | RLGPVQNE(V/I) | |
|  |  |  |  |  |  |  | VLSDFKTWL | |
| 300256 | Chronic | 1a | 3307 | 98 | 6 | 3 | (H/D)YPYRLWHY | |
|  |  |  |  |  |  |  | GP(RL/KM)GVRAT | |
|  |  |  |  |  |  |  | HP(N/S)IEEVAL | |
|  |  |  |  |  |  |  | YGKAIPLEVI | |
|  |  |  |  |  |  |  | HAVGIFRAA | |
|  |  |  |  |  |  |  | ALGVNAVAYY | |
| HOKD0485FX | Chronic | 1b | 734 | 82 | 4 | 2 | HS(KK/RR)KCDEL | |
|  |  |  |  |  |  |  | HPVTKYI(M/T)* | |
|  |  |  |  |  |  |  | STNPKPQR(Q/K) | |
|  |  |  |  |  |  |  | VTLTHPVTKY | |
| THDS1086MX | Chronic | 1a | 2115 | 99 | 4 | 2 | KLVAMG(L/I)NAV | |
|  |  |  |  |  |  |  | TLSPYYKR(Y/H)I | |
|  |  |  |  |  |  |  | ARMVMMTHF | |
|  |  |  |  |  |  |  | VRMVMMTHF | |
| THGS0684MX | Chronic | 1a | 1388 | 100 | 5 | 3 | TSILGIGT(A/V) | |
|  |  |  |  |  |  |  | SILGIGT(A/V)L | |
|  |  |  |  |  |  |  | AWETAR(H/Y)TPV | |
|  |  |  |  |  |  |  | KLVAMGINAV | |
|  |  |  |  |  |  |  | ARMVMMTHF | |
| **Total** |  |  | 10945 | 494 | 30 | 14 |  | |
| * - Epitope not used due to length criteria being 9-10mer, a - Epitopes with ≥ 25 SFU / million cells in IFN-γ ELISPOT assay | | | | | | | |  |

**Supplementary File 3. Subject 300023 relative fitness estimate, co-occurring mutations and frequency of occurrence for each reconstructed haplotype.**

| **Region** | **Time** | **Viral Load*** | **Frequency** | **Relative Fitness** | **_852_RAEAQLHAW_860_**  **_2629_KSKRTPMGF_2637_**  **Mutations** | | **Co-occurring Mutations**** | | |
| --- | --- | --- | --- | --- | --- | --- | --- | --- | --- |
| NS2 | 36DPI | 19234348 | 30.80% | 1.000 |  |  |  |  |  |
|  |  |  | 27.10% | 1.000 |  |  |  |  |  |
|  |  |  | 21.80% | 1.000 |  |  |  |  |  |
|  |  |  | 20.30% | 1.000 |  |  |  |  |  |
|  | 44DPI | 17907338 | 31.30% | 1.000 |  |  |  |  |  |
|  |  |  | 25.70% | 1.000 |  |  |  |  |  |
|  |  |  | 23.70% | 1.000 |  |  |  |  |  |
|  |  |  | 19.20% | 1.000 |  |  |  |  |  |
|  | '60DPI' | 8121396 | 86.20% | 0.462 |  |  | F921S |  |  |
|  |  |  | 13.80% | 0.462 |  |  | F921S |  |  |
|  | '74DPI' | 397185 | 27.60% | 1.000 |  |  |  |  |  |
|  |  |  | 19.70% | 1.000 |  |  |  |  |  |
|  |  |  | 17.20% | 1.000 |  |  |  |  |  |
|  |  |  | 12.30% | 1.000 |  |  |  |  |  |
|  |  |  | 5.90% | 1.000 |  |  |  |  |  |
|  |  |  | 4.20% | 1.000 |  |  |  |  |  |
|  |  |  | 3.50% | 1.000 |  |  |  |  |  |
|  |  |  | 2.60% | 1.000 |  |  |  |  |  |
|  |  |  | 2.40% | 0.536 |  |  |  |  |  |
|  |  |  | 1.80% | 0.536 |  |  |  |  |  |
|  |  |  | 1.50% | 0.536 |  |  |  |  |  |
|  |  |  | 1.10% | 0.536 |  |  |  |  |  |
|  | 135DPI | 2843176 | 63.10% | 1.000 |  |  |  |  |  |
|  |  |  | 11.90% | 0.617 | Q856H |  |  |  |  |
|  |  |  | 9.40% | 0.599 | Q856L |  |  |  |  |
|  |  |  | 8.00% | 1.000 |  |  |  |  |  |
|  |  |  | 2.70% | 0.358 | A853T |  |  |  |  |
|  |  |  | 1.90% | 0.599 | Q856L |  |  |  |  |
|  |  |  | 1.60% | 0.617 | Q856H |  |  |  |  |
|  |  |  | 1.30% | 0.599 | Q856L |  |  |  |  |
|  | 197DPI | 5896155 | 44.50% | 0.617 | Q856L |  |  |  |  |
|  |  |  | 21.00% | 0.617 | Q856L |  |  |  |  |
|  |  |  | 13.20% | 0.617 | Q856L |  |  |  |  |
|  |  |  | 8.20% | 0.617 | Q856L |  |  |  |  |
|  |  |  | 6.30% | 0.617 | Q856L |  |  |  |  |
|  |  |  | 4.20% | 0.617 | Q856L |  |  |  |  |
|  |  |  | 2.60% | 0.617 | Q856L |  |  |  |  |
| NS5B | 36DPI | 19234348 | 65.80% | 1.000 |  |  |  |  |  |
|  |  |  | 20.10% | 1.000 |  |  |  |  |  |
|  |  |  | 10.90% | 0.175 |  |  | E2866K |  |  |
|  |  |  | 3.10% | 0.175 |  |  | E2866K |  |  |
|  | 44DPI | 17907338 | 7.30% | 5.647 |  |  | H2750Q | T2917A |  |
|  |  |  | 7.00% | 1.428 |  |  | T2917A |  |  |
|  |  |  | 7.00% | 3.966 |  |  | H2750Q |  |  |
|  |  |  | 6.90% | 1.000 |  |  |  |  |  |
|  |  |  | 6.90% | 5.647 |  |  | H2750Q | T2917A |  |
|  |  |  | 6.70% | 1.000 |  |  |  |  |  |
|  |  |  | 6.60% | 3.966 |  |  | H2750Q |  |  |
|  |  |  | 6.50% | 1.428 |  |  | T2917A |  |  |
|  |  |  | 6.10% | 1.000 |  |  |  |  |  |
|  |  |  | 5.80% | 1.428 |  |  | T2917A |  |  |
|  |  |  | 5.80% | 3.966 |  |  | H2750Q |  |  |
|  |  |  | 5.70% | 1.428 |  |  | T2917A |  |  |
|  |  |  | 5.60% | 3.966 |  |  | H2750Q |  |  |
|  |  |  | 5.40% | 5.647 |  |  | H2750Q | T2917A |  |
|  |  |  | 5.30% | 5.647 |  |  | H2750Q | T2917A |  |
|  |  |  | 5.30% | 1.000 |  |  |  |  |  |
|  | 60DPI | 8121396 | 83.60% | 1.000 |  |  |  |  |  |
|  |  |  | 3.90% | 1.000 |  |  |  |  |  |
|  |  |  | 3.80% | 1.428 |  |  | T2917A |  |  |
|  |  |  | 3.60% | 1.000 |  |  |  |  |  |
|  |  |  | 3.40% | 1.000 |  |  |  |  |  |
|  |  |  | 1.70% | 3.966 |  |  | H2750Q |  |  |
|  | 74DPI | 397185 | 8.90% | 1.000 |  |  |  |  |  |
|  |  |  | 8.20% | 1.000 |  |  | T2917A |  |  |
|  |  |  | 8.20% | 1.428 |  |  |  |  |  |
|  |  |  | 7.40% | 3.966 |  |  | H2750Q |  |  |
|  |  |  | 6.80% | 1.428 |  |  | T2917A |  |  |
|  |  |  | 6.70% | 1.000 |  |  |  |  |  |
|  |  |  | 6.60% | 3.966 |  |  | H2750Q |  |  |
|  |  |  | 6.00% | 1.000 |  |  |  |  |  |
|  |  |  | 6.00% | 1.428 |  |  | T2917A |  |  |
|  |  |  | 5.70% | 5.647 |  |  | H2750Q | T2917A |  |
|  |  |  | 5.50% | 3.966 |  |  | H2750Q |  |  |
|  |  |  | 5.40% | 5.647 |  |  | H2750Q | T2917A |  |
|  |  |  | 5.00% | 1.428 |  |  | T2917A |  |  |
|  |  |  | 4.90% | 5.647 |  |  | H2750Q | T2917A |  |
|  |  |  | 4.40% | 3.966 |  |  | H2750Q |  |  |
|  |  |  | 4.20% | 5.647 |  |  | H2750Q | T2917A |  |
|  | 135DPI | 2843176 | 43.60% | 0.337 | K2629N |  |  |  |  |
|  |  |  | 21.00% | 0.337 | K2629N |  |  |  |  |
|  |  |  | 17.20% | 0.337 | K2629N |  |  |  |  |
|  |  |  | 7.90% | 0.337 | K2629N |  |  |  |  |
|  |  |  | 3.50% | 0.060 | K2629N |  | T2930S |  |  |
|  |  |  | 2.10% | 1.000 |  |  |  |  |  |
|  |  |  | 2.00% | 0.141 | K2629N |  |  |  |  |
|  |  |  | 1.60% | 0.060 | K2629N |  | T2930S |  |  |
|  |  |  | 1.20% | 0.146 | K2629N | R2632G |  |  |  |
|  | 197DPI | 5896155 | 97.90% | 0.337 | K2629N |  |  |  |  |
|  |  |  | 1.00% | 0.171 | K2629N |  | R2690K |  |  |
|  |  |  | 1.00% | 0.055 | K2629N |  | D2772E |  |  |
| *Viral Load measured in IU/ML. | | | | | | | | | |
| **Only non-synonymous mutations are shown. | | | | | | | | | |

**Supplementary File 4. Subject 300240 relative fitness estimate, co-occurring mutations and frequency of occurrence for each reconstructed haplotype.**

| **Region** | **Time** | **Viral Load*** | **Frequency** | **Relative Fitness** | **_1602_RAQAPPPSW_1610_**  **_1633_RLGPVQNEV_1641_**  **Mutations** | | **Co-occurring Mutations**** | | | |  |
| --- | --- | --- | --- | --- | --- | --- | --- | --- | --- | --- | --- |
| NS3 | 44DPI | 54887 | 18.50% | 1.0000 |  |  |  |  |  | |  |
|  |  |  | 13.20% | 1.0000 |  |  |  |  |  | |  |
|  |  |  | 8.40% | 1.0000 |  |  |  |  |  | |  |
|  |  |  | 6.00% | 1.0000 |  |  |  |  |  | |  |
|  |  |  | 5.60% | 1.0000 |  |  |  |  |  | |  |
|  |  |  | 5.10% | 1.0000 |  |  |  |  |  | |  |
|  |  |  | 4.80% | 1.0000 |  |  |  |  |  | |  |
|  |  |  | 3.80% | 1.0000 |  |  |  |  |  | |  |
|  |  |  | 3.30% | 1.0000 |  |  |  |  |  | |  |
|  |  |  | 3.20% | 0.4132 |  |  | T1411I |  |  | |  |
|  |  |  | 3.00% | 1.0000 |  |  |  |  |  | |  |
|  |  |  | 2.80% | 1.0000 |  |  |  |  |  | |  |
|  |  |  | 2.50% | 1.0000 |  |  |  |  |  | |  |
|  |  |  | 2.40% | 0.4132 |  |  | T1411I |  |  | |  |
|  |  |  | 2.30% | 1.0000 |  |  |  |  |  | |  |
|  |  |  | 2.20% | 1.0000 |  |  |  |  |  | |  |
|  |  |  | 2.10% | 1.0000 |  |  |  |  |  | |  |
|  |  |  | 1.70% | 1.0000 |  |  |  |  |  | |  |
|  |  |  | 1.60% | 1.0000 |  |  |  |  |  | |  |
|  |  |  | 1.60% | 0.4132 |  |  | T1411I |  |  | |  |
|  |  |  | 1.60% | 1.0000 |  |  |  |  |  | |  |
|  |  |  | 1.60% | 1.0000 |  |  |  |  |  | |  |
|  |  |  | 1.40% | 1.0000 |  |  |  |  |  | |  |
|  |  |  | 1.40% | 1.0000 |  |  |  |  |  | |  |
|  | 57DPI | 85473 | 53.70% | 1.0000 |  |  |  |  |  | |  |
|  |  |  | 23.90% | 0.4132 |  |  | T1411I |  |  | |  |
|  |  |  | 12.60% | 3.5094 |  |  | V1202I |  |  | |  |
|  |  |  | 6.10% | 1.4445 |  |  | V1202I | T1411I |  | |  |
|  |  |  | 2.60% | 0.0107 |  |  | A1576G |  |  | |  |
|  |  |  | 1.10% | 0.0045 |  |  | A1576G | T1411I |  | |  |
|  | 220DPI | 44449 | 96.60% | 0.0107 | P1606L |  |  | T1509N |  | |  |
|  |  |  | 1.70% | 0.0107 | P1606L |  |  | T1509K |  | |  |
|  |  |  | 1.70% | 0.0016 | P1606L |  | R1395G | T1509N |  | |  |
|  | 538DPI | 62174 | 37.30% | 0.0031 | P1606L | V1641I |  |  |  | |  |
|  |  |  | 35.50% | 0.0031 | P1606L | V1641I |  |  |  | |  |
|  |  |  | 12.60% | 0.0031 | P1606L | V1641I |  |  |  | |  |
|  |  |  | 10.60% | 0.0031 | P1606L | V1641I |  |  |  | |  |
|  |  |  | 2.10% | 0.0000 | P1606L | V1641I |  |  |  | |  |
|  |  |  | 1.80% | 0.0000 | P1606L | V1641I |  |  |  | |  |
| *Viral Load measured in IU/ML. | | | | | | | | | |  | |
| **Only non-synonymous mutations are shown. | | | | | | | | | |  | |

**Supplementary File 5 - Subject 300256 relative fitness estimate, co-occurring mutations and frequency of occurrence for each reconstructed haplotype.**

| **Region** | **Time** | **Viral Load*** | **Frequency** | **Relative Fitness** | **_1359_HPNIEEVAL_1367_ Mutations** | **Co-occurring Mutations**** | | |
| --- | --- | --- | --- | --- | --- | --- | --- | --- |
| NS3 | 44DPI | 34149824 | 27.20% | 1.000 |  |  |  |  |
|  |  |  | 8.30% | 1.000 |  |  |  |  |
|  |  |  | 7.30% | 0.151 |  |  |  |  |
|  |  |  | 7.10% | 1.000 |  |  |  |  |
|  |  |  | 6.90% | 1.000 |  |  |  |  |
|  |  |  | 5.70% | 1.000 |  |  |  |  |
|  |  |  | 4.40% | 1.000 |  |  |  |  |
|  |  |  | 4.20% | 0.008 |  | V1109I |  |  |
|  |  |  | 3.90% | 0.151 |  |  |  |  |
|  |  |  | 3.80% | 1.000 |  |  |  |  |
|  |  |  | 3.70% | 0.001 |  | V1109I |  |  |
|  |  |  | 3.60% | 1.000 |  |  |  |  |
|  |  |  | 3.60% | 1.000 |  |  |  |  |
|  |  |  | 2.70% | 1.000 |  |  |  |  |
|  |  |  | 2.60% | 1.000 |  |  |  |  |
|  |  |  | 2.50% | 1.000 |  |  |  |  |
|  |  |  | 2.30% | 1.000 |  |  |  |  |
|  | 58DPI | 19188762 | 51.70% | 0.330 |  | V1109I |  |  |
|  |  |  | 14.30% | 0.330 |  | V1109I |  |  |
|  |  |  | 11.30% | 0.330 |  | V1109I |  |  |
|  |  |  | 10.30% | 0.330 |  | V1109I |  |  |
|  |  |  | 5.50% | 0.049 |  | V1109I | M1268V |  |
|  |  |  | 3.70% | 0.330 |  | V1109I |  |  |
|  |  |  | 3.20% | 0.330 |  | V1109I |  |  |
|  | 79DPI | 812622 | 75.70% | 0.330 |  | V1109I |  |  |
|  |  |  | 7.30% | 0.330 |  | V1109I |  |  |
|  |  |  | 6.40% | 0.330 |  | V1109I |  |  |
|  |  |  | 5.10% | 0.330 |  | V1109I |  |  |
|  |  |  | 3.00% | 0.050 |  | V1109I | Y1249C |  |
|  |  |  | 1.40% | 0.035 |  | V1109I | C1594Y |  |
|  |  |  | 1.10% | 0.050 |  | V1109I | H1272R |  |
|  | 96DPI | 50774 | 100.00% | 0.330 |  | V1109I |  |  |
|  | 286DPI | 14853 | 73.90% | 0.031 | N1361S | V1109I | T1408I |  |
|  |  |  | 16.40% | 0.007 | N1361S | V1109I | T1408I | M1646T |
|  |  |  | 3.60% | 0.031 | N1361S | V1109I | T1408I |  |
|  |  |  | 3.60% | 0.004 | N1361S | V1109I | T1408I |  |
|  |  |  | 2.50% | 0.031 | N1361S | V1109I | T1408I |  |
| *Viral Load measured in IU/ML. | | | | | | | |  |
| **Only non-synonymous mutations are shown. | | | | | | | |  |

**Supplementary File 6. Subject HOKD0485FX relative fitness estimate, co-occurring mutations and frequency of occurrence for each reconstructed haplotype.**

| **Region** | **Time** | **Viral Load*** | **Frequency** | **Relative Fitness** | **_1395_HSKKKCDEL_1403_**  **Mutations** | | **Co-occurring Mutations**** | | |
| --- | --- | --- | --- | --- | --- | --- | --- | --- | --- |
| NS3 | 30DPI | 733849 | 13.10% | 1.0000 |  |  |  |  |  |
|  |  |  | 13.00% | 0.1509 |  |  |  | D1605G |  |
|  |  |  | 11.70% | 0.1509 |  |  |  | C1518F |  |
|  |  |  | 9.80% | 0.1509 |  |  |  | D1605G |  |
|  |  |  | 5.10% | 0.1509 |  |  |  | D1605G |  |
|  |  |  | 5.10% | 1.0000 |  |  |  |  |  |
|  |  |  | 4.90% | 0.1509 |  |  |  | C1518F |  |
|  |  |  | 4.50% | 1.0000 |  |  |  |  |  |
|  |  |  | 4.00% | 1.0000 |  |  |  |  |  |
|  |  |  | 3.60% | 1.0000 |  |  |  |  |  |
|  |  |  | 2.30% | 0.0336 |  |  |  | A1230T | A1302T |
|  |  |  | 2.20% | 0.0144 |  |  |  | S1536P |  |
|  |  |  | 2.10% | 0.1511 |  |  |  | T1286P |  |
|  |  |  | 1.60% | 0.1199 |  |  |  |  |  |
|  |  |  | 1.60% | 0.0350 |  |  |  | V1198A | I1314M |
|  |  |  | 1.60% | 0.0228 |  |  |  | V1198A |  |
|  |  |  | 1.40% | 0.1509 |  |  |  | P1241S | A1302T |
|  |  |  | 1.20% | 0.1509 |  |  |  | G1233D |  |
|  | 72DPI | 175219 | 66.90% | 0.2373 | K1398R |  |  |  |  |
|  |  |  | 7.50% | 0.2373 | K1398R |  |  |  |  |
|  |  |  | 7.20% | 0.2373 | K1398R |  |  |  |  |
|  |  |  | 6.40% | 0.0358 | K1398R |  |  | G1307E |  |
|  |  |  | 3.10% | 0.0364 | K1398R |  |  | S1215F |  |
|  |  |  | 2.60% | 0.2373 | K1398R |  |  |  |  |
|  |  |  | 2.40% | 0.0359 | K1398R |  | Y1644C |  |  |
|  |  |  | 2.20% | 0.2373 | K1398R |  |  |  |  |
|  |  |  | 1.60% | 0.0245 | K1398R |  |  | T1459I |  |
|  | 79DPI | 44452 | 36.70% | 0.2373 | K1398R |  |  |  |  |
|  |  |  | 23.80% | 0.2373 | K1398R |  |  |  |  |
|  |  |  | 23.80% | 0.2373 | K1398R |  |  |  |  |
|  |  |  | 15.70% | 0.2373 | K1398R |  |  |  |  |
|  | 93DPI | 407392 | 41.30% | 0.3420 | K1398R |  | V1641I |  |  |
|  |  |  | 27.30% | 0.0324 | K1398R |  | V1641I | A1113T |  |
|  |  |  | 11.20% | 0.0516 | K1398R |  | V1641I | A1085V |  |
|  |  |  | 7.40% | 0.0049 | K1398R |  | V1641I | A1085V | A1113T |
|  |  |  | 3.40% | 0.3420 | K1398R |  | V1641I |  |  |
|  |  |  | 2.30% | 0.0324 | K1398R |  | V1641I | A1113T |  |
|  |  |  | 2.10% | 0.2373 | K1398R |  |  |  |  |
|  |  |  | 1.70% | 0.1189 | K1398R |  | V1641I | R1496G |  |
|  |  |  | 1.60% | 0.3420 | K1398R |  | V1641I |  |  |
|  |  |  | 1.50% | 0.0223 | K1398R |  | V1641I | A1113T |  |
|  | 107DPI | 24969 | 100.00% | 0.0039 | D1401N |  | V1641I |  |  |
|  | 121DPI | 77723 | 19.00% | 0.0034 | K1398R | K1397R | V1641A |  |  |
|  |  |  | 11.80% | 0.0034 | K1398R | K1397R | V1641A |  |  |
|  |  |  | 10.80% | 0.0034 | K1398R | K1397R | V1641A |  |  |
|  |  |  | 7.50% | 0.0034 | K1398R | K1397R | V1641A |  |  |
|  |  |  | 5.90% | 0.0034 | K1398R | K1397R |  |  |  |
|  |  |  | 4.30% | 0.0285 | K1398R | K1397R | V1641A |  |  |
|  |  |  | 4.30% | 0.0034 | K1398R | K1397R | V1641A |  |  |
|  |  |  | 4.00% | 0.0034 | K1398R | K1397R | V1641A |  |  |
|  |  |  | 3.10% | 0.0295 | K1398R |  | V1641A |  |  |
|  |  |  | 2.90% | 0.0148 | K1398R | K1397R | V1641A |  |  |
|  |  |  | 2.60% | 0.0005 | K1398R | K1397R | V1641A | I1285V |  |
|  |  |  | 2.50% | 0.0285 | K1398R | K1397R |  |  |  |
|  |  |  | 2.40% | 0.0285 | K1398R | K1397R |  |  |  |
|  |  |  | 2.30% | 0.0073 | K1398R | K1397R | V1641T |  |  |
|  |  |  | 2.00% | 0.0034 | K1398R | K1397R | V1641A |  |  |
|  |  |  | 2.00% | 0.0034 | K1398R | K1397R | V1641A |  |  |
|  |  |  | 2.00% | 0.0295 |  | K1397R | V1641A |  |  |
|  |  |  | 1.90% | 0.0295 |  | K1397R | V1641A |  |  |
|  |  |  | 1.90% | 0.0005 | K1398R | K1397R | V1641A | A1405V |  |
|  |  |  | 1.80% | 0.0148 | K1398R |  | V1641A |  |  |
|  |  |  | 1.80% | 0.0005 | K1398R | K1397R | V1641A | I1285V |  |
|  |  |  | 1.70% | 0.0148 | K1398R |  | V1641A |  |  |
|  |  |  | 1.70% | 0.0073 | K1398R | K1397R | V1641T |  |  |
|  | 149DPI | 254245 | 97.10% | 0.0031 | K1398R | K1397R | M1646T |  |  |
|  |  |  | 1.80% | 0.0005 | K1398R | K1397R | M1646T | A1597T |  |
|  |  |  | 1.10% | 0.0005 | K1398R | K1397R | M1646T | G1489D |  |
|  | 233DPI | 350658 | 62.00% | 0.0002 | K1398R | K1397R | M1646T |  |  |
|  |  |  | 12.00% | 0.0002 | K1398R | K1397R | M1646T |  |  |
|  |  |  | 3.60% | 0.0002 | K1398R | K1397R | M1646T | A1647T |  |
|  |  |  | 3.50% | 0.0002 | K1398R | K1397R | M1646T |  |  |
|  |  |  | 3.20% | 0.0000 | K1398R | K1397R | M1646T | K1162R |  |
|  |  |  | 3.10% | 0.0002 | K1398R | K1397R | M1646T |  |  |
|  |  |  | 2.70% | 0.0015 | K1398R | K1397R | M1646T | V1329I |  |
|  |  |  | 2.70% | 0.0003 | K1398R | K1397R | M1646T | V1641I |  |
|  |  |  | 2.60% | 0.0002 | K1398R | K1397R | M1646T |  |  |
|  |  |  | 2.50% | 0.0002 | K1398R | K1397R | M1646T |  |  |
|  |  |  | 2.20% | 0.0002 | K1398R | K1397R | M1646T | K1088R |  |
| *Viral Load measured in IU/ML. | | | | | | | | |  |
| **Only non-synonymous mutations are shown. | | | | | | | | |  |
