# Supplementary material for: Temporal dynamics of viral fitness and the adaptive immune response in HCV infection
Source: eLife. 2025 Aug 29;13:RP102232. doi: 10.7554/eLife.102232 (PMC12396813; doi:10.7554/eLife.102232)
Supplement: Supplementary file 2. [file elife-102232-supp2.docx]

**Supplementary File 2 – Summary of epitope selection and positive (IFN-γ) ELISPOT assay responses in subjects who developed chronic infection.**

| **Subject ID** | **Disease Outcome** | **Genotype** | **No. of potential Epitopes** | **No. of selected Epitopes** | **No. of positive IFN-γ ELISPOT** | **No. of epitopes underwent escape** | **Epitope ^a^** | |
| --- | --- | --- | --- | --- | --- | --- | --- | --- |
| 300023 | Chronic | 1a | 2213 | 45 | 8 | 2 | (K/N)SKRTPMGF | |
|  |  |  |  |  |  |  | RAEA(Q/H)LHAW | |
|  |  |  |  |  |  |  | CINGVCWTV | |
|  |  |  |  |  |  |  | VLSDFKTWL | |
|  |  |  |  |  |  |  | AEVIAPAVQT | |
|  |  |  |  |  |  |  | FAWYLKGKW | |
|  |  |  |  |  |  |  | AELIEANLLW | |
|  |  |  |  |  |  |  | WLGNIIMFA | |
| 300240 | Chronic | 3a | 1188 | 70 | 3 | 2 | RAQA(P/L)PPSW | |
|  |  |  |  |  |  |  | RLGPVQNE(V/I) | |
|  |  |  |  |  |  |  | VLSDFKTWL | |
| 300256 | Chronic | 1a | 3307 | 98 | 6 | 3 | (H/D)YPYRLWHY | |
|  |  |  |  |  |  |  | GP(RL/KM)GVRAT | |
|  |  |  |  |  |  |  | HP(N/S)IEEVAL | |
|  |  |  |  |  |  |  | YGKAIPLEVI | |
|  |  |  |  |  |  |  | HAVGIFRAA | |
|  |  |  |  |  |  |  | ALGVNAVAYY | |
| HOKD0485FX | Chronic | 1b | 734 | 82 | 4 | 2 | HS(KK/RR)KCDEL | |
|  |  |  |  |  |  |  | HPVTKYI(M/T)* | |
|  |  |  |  |  |  |  | STNPKPQR(Q/K) | |
|  |  |  |  |  |  |  | VTLTHPVTKY | |
| THDS1086MX | Chronic | 1a | 2115 | 99 | 4 | 2 | KLVAMG(L/I)NAV | |
|  |  |  |  |  |  |  | TLSPYYKR(Y/H)I | |
|  |  |  |  |  |  |  | ARMVMMTHF | |
|  |  |  |  |  |  |  | VRMVMMTHF | |
| THGS0684MX | Chronic | 1a | 1388 | 100 | 5 | 3 | TSILGIGT(A/V) | |
|  |  |  |  |  |  |  | SILGIGT(A/V)L | |
|  |  |  |  |  |  |  | AWETAR(H/Y)TPV | |
|  |  |  |  |  |  |  | KLVAMGINAV | |
|  |  |  |  |  |  |  | ARMVMMTHF | |
| **Total** |  |  | 10945 | 494 | 30 | 14 |  | |
| * - Epitope not used due to length criteria being 9-10mer, a - Epitopes with ≥ 25 SFU / million cells in IFN-γ ELISPOT assay | | | | | | | |  |
