# Supplementary material for: Temporal dynamics of viral fitness and the adaptive immune response in HCV infection
Source: eLife. 2025 Aug 29;13:RP102232. doi: 10.7554/eLife.102232 (PMC12396813; doi:10.7554/eLife.102232)
Supplement: Supplementary file 3. [file elife-102232-supp3.docx]

**Supplementary File 3. Subject 300023 relative fitness estimate, co-occurring mutations and frequency of occurrence for each reconstructed haplotype.**

| **Region** | **Time** | **Viral Load*** | **Frequency** | **Relative Fitness** | **_852_RAEAQLHAW_860_**  **_2629_KSKRTPMGF_2637_**  **Mutations** | | **Co-occurring Mutations**** | | |
| --- | --- | --- | --- | --- | --- | --- | --- | --- | --- |
| NS2 | 36DPI | 19234348 | 30.80% | 1.000 |  |  |  |  |  |
|  |  |  | 27.10% | 1.000 |  |  |  |  |  |
|  |  |  | 21.80% | 1.000 |  |  |  |  |  |
|  |  |  | 20.30% | 1.000 |  |  |  |  |  |
|  | 44DPI | 17907338 | 31.30% | 1.000 |  |  |  |  |  |
|  |  |  | 25.70% | 1.000 |  |  |  |  |  |
|  |  |  | 23.70% | 1.000 |  |  |  |  |  |
|  |  |  | 19.20% | 1.000 |  |  |  |  |  |
|  | '60DPI' | 8121396 | 86.20% | 0.462 |  |  | F921S |  |  |
|  |  |  | 13.80% | 0.462 |  |  | F921S |  |  |
|  | '74DPI' | 397185 | 27.60% | 1.000 |  |  |  |  |  |
|  |  |  | 19.70% | 1.000 |  |  |  |  |  |
|  |  |  | 17.20% | 1.000 |  |  |  |  |  |
|  |  |  | 12.30% | 1.000 |  |  |  |  |  |
|  |  |  | 5.90% | 1.000 |  |  |  |  |  |
|  |  |  | 4.20% | 1.000 |  |  |  |  |  |
|  |  |  | 3.50% | 1.000 |  |  |  |  |  |
|  |  |  | 2.60% | 1.000 |  |  |  |  |  |
|  |  |  | 2.40% | 0.536 |  |  |  |  |  |
|  |  |  | 1.80% | 0.536 |  |  |  |  |  |
|  |  |  | 1.50% | 0.536 |  |  |  |  |  |
|  |  |  | 1.10% | 0.536 |  |  |  |  |  |
|  | 135DPI | 2843176 | 63.10% | 1.000 |  |  |  |  |  |
|  |  |  | 11.90% | 0.617 | Q856H |  |  |  |  |
|  |  |  | 9.40% | 0.599 | Q856L |  |  |  |  |
|  |  |  | 8.00% | 1.000 |  |  |  |  |  |
|  |  |  | 2.70% | 0.358 | A853T |  |  |  |  |
|  |  |  | 1.90% | 0.599 | Q856L |  |  |  |  |
|  |  |  | 1.60% | 0.617 | Q856H |  |  |  |  |
|  |  |  | 1.30% | 0.599 | Q856L |  |  |  |  |
|  | 197DPI | 5896155 | 44.50% | 0.617 | Q856L |  |  |  |  |
|  |  |  | 21.00% | 0.617 | Q856L |  |  |  |  |
|  |  |  | 13.20% | 0.617 | Q856L |  |  |  |  |
|  |  |  | 8.20% | 0.617 | Q856L |  |  |  |  |
|  |  |  | 6.30% | 0.617 | Q856L |  |  |  |  |
|  |  |  | 4.20% | 0.617 | Q856L |  |  |  |  |
|  |  |  | 2.60% | 0.617 | Q856L |  |  |  |  |
| NS5B | 36DPI | 19234348 | 65.80% | 1.000 |  |  |  |  |  |
|  |  |  | 20.10% | 1.000 |  |  |  |  |  |
|  |  |  | 10.90% | 0.175 |  |  | E2866K |  |  |
|  |  |  | 3.10% | 0.175 |  |  | E2866K |  |  |
|  | 44DPI | 17907338 | 7.30% | 5.647 |  |  | H2750Q | T2917A |  |
|  |  |  | 7.00% | 1.428 |  |  | T2917A |  |  |
|  |  |  | 7.00% | 3.966 |  |  | H2750Q |  |  |
|  |  |  | 6.90% | 1.000 |  |  |  |  |  |
|  |  |  | 6.90% | 5.647 |  |  | H2750Q | T2917A |  |
|  |  |  | 6.70% | 1.000 |  |  |  |  |  |
|  |  |  | 6.60% | 3.966 |  |  | H2750Q |  |  |
|  |  |  | 6.50% | 1.428 |  |  | T2917A |  |  |
|  |  |  | 6.10% | 1.000 |  |  |  |  |  |
|  |  |  | 5.80% | 1.428 |  |  | T2917A |  |  |
|  |  |  | 5.80% | 3.966 |  |  | H2750Q |  |  |
|  |  |  | 5.70% | 1.428 |  |  | T2917A |  |  |
|  |  |  | 5.60% | 3.966 |  |  | H2750Q |  |  |
|  |  |  | 5.40% | 5.647 |  |  | H2750Q | T2917A |  |
|  |  |  | 5.30% | 5.647 |  |  | H2750Q | T2917A |  |
|  |  |  | 5.30% | 1.000 |  |  |  |  |  |
|  | 60DPI | 8121396 | 83.60% | 1.000 |  |  |  |  |  |
|  |  |  | 3.90% | 1.000 |  |  |  |  |  |
|  |  |  | 3.80% | 1.428 |  |  | T2917A |  |  |
|  |  |  | 3.60% | 1.000 |  |  |  |  |  |
|  |  |  | 3.40% | 1.000 |  |  |  |  |  |
|  |  |  | 1.70% | 3.966 |  |  | H2750Q |  |  |
|  | 74DPI | 397185 | 8.90% | 1.000 |  |  |  |  |  |
|  |  |  | 8.20% | 1.000 |  |  | T2917A |  |  |
|  |  |  | 8.20% | 1.428 |  |  |  |  |  |
|  |  |  | 7.40% | 3.966 |  |  | H2750Q |  |  |
|  |  |  | 6.80% | 1.428 |  |  | T2917A |  |  |
|  |  |  | 6.70% | 1.000 |  |  |  |  |  |
|  |  |  | 6.60% | 3.966 |  |  | H2750Q |  |  |
|  |  |  | 6.00% | 1.000 |  |  |  |  |  |
|  |  |  | 6.00% | 1.428 |  |  | T2917A |  |  |
|  |  |  | 5.70% | 5.647 |  |  | H2750Q | T2917A |  |
|  |  |  | 5.50% | 3.966 |  |  | H2750Q |  |  |
|  |  |  | 5.40% | 5.647 |  |  | H2750Q | T2917A |  |
|  |  |  | 5.00% | 1.428 |  |  | T2917A |  |  |
|  |  |  | 4.90% | 5.647 |  |  | H2750Q | T2917A |  |
|  |  |  | 4.40% | 3.966 |  |  | H2750Q |  |  |
|  |  |  | 4.20% | 5.647 |  |  | H2750Q | T2917A |  |
|  | 135DPI | 2843176 | 43.60% | 0.337 | K2629N |  |  |  |  |
|  |  |  | 21.00% | 0.337 | K2629N |  |  |  |  |
|  |  |  | 17.20% | 0.337 | K2629N |  |  |  |  |
|  |  |  | 7.90% | 0.337 | K2629N |  |  |  |  |
|  |  |  | 3.50% | 0.060 | K2629N |  | T2930S |  |  |
|  |  |  | 2.10% | 1.000 |  |  |  |  |  |
|  |  |  | 2.00% | 0.141 | K2629N |  |  |  |  |
|  |  |  | 1.60% | 0.060 | K2629N |  | T2930S |  |  |
|  |  |  | 1.20% | 0.146 | K2629N | R2632G |  |  |  |
|  | 197DPI | 5896155 | 97.90% | 0.337 | K2629N |  |  |  |  |
|  |  |  | 1.00% | 0.171 | K2629N |  | R2690K |  |  |
|  |  |  | 1.00% | 0.055 | K2629N |  | D2772E |  |  |
| *Viral Load measured in IU/ML. | | | | | | | | | |
| **Only non-synonymous mutations are shown. | | | | | | | | | |
