# Supplementary material for: Temporal dynamics of viral fitness and the adaptive immune response in HCV infection
Source: eLife. 2025 Aug 29;13:RP102232. doi: 10.7554/eLife.102232 (PMC12396813; doi:10.7554/eLife.102232)
Supplement: Supplementary file 4. [file elife-102232-supp4.docx]

**Supplementary File 4. Subject 300240 relative fitness estimate, co-occurring mutations and frequency of occurrence for each reconstructed haplotype.**

| **Region** | **Time** | **Viral Load*** | **Frequency** | **Relative Fitness** | **_1602_RAQAPPPSW_1610_**  **_1633_RLGPVQNEV_1641_**  **Mutations** | | **Co-occurring Mutations**** | | | |  |
| --- | --- | --- | --- | --- | --- | --- | --- | --- | --- | --- | --- |
| NS3 | 44DPI | 54887 | 18.50% | 1.0000 |  |  |  |  |  | |  |
|  |  |  | 13.20% | 1.0000 |  |  |  |  |  | |  |
|  |  |  | 8.40% | 1.0000 |  |  |  |  |  | |  |
|  |  |  | 6.00% | 1.0000 |  |  |  |  |  | |  |
|  |  |  | 5.60% | 1.0000 |  |  |  |  |  | |  |
|  |  |  | 5.10% | 1.0000 |  |  |  |  |  | |  |
|  |  |  | 4.80% | 1.0000 |  |  |  |  |  | |  |
|  |  |  | 3.80% | 1.0000 |  |  |  |  |  | |  |
|  |  |  | 3.30% | 1.0000 |  |  |  |  |  | |  |
|  |  |  | 3.20% | 0.4132 |  |  | T1411I |  |  | |  |
|  |  |  | 3.00% | 1.0000 |  |  |  |  |  | |  |
|  |  |  | 2.80% | 1.0000 |  |  |  |  |  | |  |
|  |  |  | 2.50% | 1.0000 |  |  |  |  |  | |  |
|  |  |  | 2.40% | 0.4132 |  |  | T1411I |  |  | |  |
|  |  |  | 2.30% | 1.0000 |  |  |  |  |  | |  |
|  |  |  | 2.20% | 1.0000 |  |  |  |  |  | |  |
|  |  |  | 2.10% | 1.0000 |  |  |  |  |  | |  |
|  |  |  | 1.70% | 1.0000 |  |  |  |  |  | |  |
|  |  |  | 1.60% | 1.0000 |  |  |  |  |  | |  |
|  |  |  | 1.60% | 0.4132 |  |  | T1411I |  |  | |  |
|  |  |  | 1.60% | 1.0000 |  |  |  |  |  | |  |
|  |  |  | 1.60% | 1.0000 |  |  |  |  |  | |  |
|  |  |  | 1.40% | 1.0000 |  |  |  |  |  | |  |
|  |  |  | 1.40% | 1.0000 |  |  |  |  |  | |  |
|  | 57DPI | 85473 | 53.70% | 1.0000 |  |  |  |  |  | |  |
|  |  |  | 23.90% | 0.4132 |  |  | T1411I |  |  | |  |
|  |  |  | 12.60% | 3.5094 |  |  | V1202I |  |  | |  |
|  |  |  | 6.10% | 1.4445 |  |  | V1202I | T1411I |  | |  |
|  |  |  | 2.60% | 0.0107 |  |  | A1576G |  |  | |  |
|  |  |  | 1.10% | 0.0045 |  |  | A1576G | T1411I |  | |  |
|  | 220DPI | 44449 | 96.60% | 0.0107 | P1606L |  |  | T1509N |  | |  |
|  |  |  | 1.70% | 0.0107 | P1606L |  |  | T1509K |  | |  |
|  |  |  | 1.70% | 0.0016 | P1606L |  | R1395G | T1509N |  | |  |
|  | 538DPI | 62174 | 37.30% | 0.0031 | P1606L | V1641I |  |  |  | |  |
|  |  |  | 35.50% | 0.0031 | P1606L | V1641I |  |  |  | |  |
|  |  |  | 12.60% | 0.0031 | P1606L | V1641I |  |  |  | |  |
|  |  |  | 10.60% | 0.0031 | P1606L | V1641I |  |  |  | |  |
|  |  |  | 2.10% | 0.0000 | P1606L | V1641I |  |  |  | |  |
|  |  |  | 1.80% | 0.0000 | P1606L | V1641I |  |  |  | |  |
| *Viral Load measured in IU/ML. | | | | | | | | | |  | |
| **Only non-synonymous mutations are shown. | | | | | | | | | |  | |
