# Supplementary material for: Temporal dynamics of viral fitness and the adaptive immune response in HCV infection
Source: eLife. 2025 Aug 29;13:RP102232. doi: 10.7554/eLife.102232 (PMC12396813; doi:10.7554/eLife.102232)
Supplement: Supplementary file 5. [file elife-102232-supp5.docx]

**Supplementary File 5 - Subject 300256 relative fitness estimate, co-occurring mutations and frequency of occurrence for each reconstructed haplotype.**

| **Region** | **Time** | **Viral Load*** | **Frequency** | **Relative Fitness** | **_1359_HPNIEEVAL_1367_ Mutations** | **Co-occurring Mutations**** | | |
| --- | --- | --- | --- | --- | --- | --- | --- | --- |
| NS3 | 44DPI | 34149824 | 27.20% | 1.000 |  |  |  |  |
|  |  |  | 8.30% | 1.000 |  |  |  |  |
|  |  |  | 7.30% | 0.151 |  |  |  |  |
|  |  |  | 7.10% | 1.000 |  |  |  |  |
|  |  |  | 6.90% | 1.000 |  |  |  |  |
|  |  |  | 5.70% | 1.000 |  |  |  |  |
|  |  |  | 4.40% | 1.000 |  |  |  |  |
|  |  |  | 4.20% | 0.008 |  | V1109I |  |  |
|  |  |  | 3.90% | 0.151 |  |  |  |  |
|  |  |  | 3.80% | 1.000 |  |  |  |  |
|  |  |  | 3.70% | 0.001 |  | V1109I |  |  |
|  |  |  | 3.60% | 1.000 |  |  |  |  |
|  |  |  | 3.60% | 1.000 |  |  |  |  |
|  |  |  | 2.70% | 1.000 |  |  |  |  |
|  |  |  | 2.60% | 1.000 |  |  |  |  |
|  |  |  | 2.50% | 1.000 |  |  |  |  |
|  |  |  | 2.30% | 1.000 |  |  |  |  |
|  | 58DPI | 19188762 | 51.70% | 0.330 |  | V1109I |  |  |
|  |  |  | 14.30% | 0.330 |  | V1109I |  |  |
|  |  |  | 11.30% | 0.330 |  | V1109I |  |  |
|  |  |  | 10.30% | 0.330 |  | V1109I |  |  |
|  |  |  | 5.50% | 0.049 |  | V1109I | M1268V |  |
|  |  |  | 3.70% | 0.330 |  | V1109I |  |  |
|  |  |  | 3.20% | 0.330 |  | V1109I |  |  |
|  | 79DPI | 812622 | 75.70% | 0.330 |  | V1109I |  |  |
|  |  |  | 7.30% | 0.330 |  | V1109I |  |  |
|  |  |  | 6.40% | 0.330 |  | V1109I |  |  |
|  |  |  | 5.10% | 0.330 |  | V1109I |  |  |
|  |  |  | 3.00% | 0.050 |  | V1109I | Y1249C |  |
|  |  |  | 1.40% | 0.035 |  | V1109I | C1594Y |  |
|  |  |  | 1.10% | 0.050 |  | V1109I | H1272R |  |
|  | 96DPI | 50774 | 100.00% | 0.330 |  | V1109I |  |  |
|  | 286DPI | 14853 | 73.90% | 0.031 | N1361S | V1109I | T1408I |  |
|  |  |  | 16.40% | 0.007 | N1361S | V1109I | T1408I | M1646T |
|  |  |  | 3.60% | 0.031 | N1361S | V1109I | T1408I |  |
|  |  |  | 3.60% | 0.004 | N1361S | V1109I | T1408I |  |
|  |  |  | 2.50% | 0.031 | N1361S | V1109I | T1408I |  |
| *Viral Load measured in IU/ML. | | | | | | | |  |
| **Only non-synonymous mutations are shown. | | | | | | | |  |
