# Supplementary material for: Temporal dynamics of viral fitness and the adaptive immune response in HCV infection
Source: eLife. 2025 Aug 29;13:RP102232. doi: 10.7554/eLife.102232 (PMC12396813; doi:10.7554/eLife.102232)
Supplement: Supplementary file 6. [file elife-102232-supp6.docx]

**Supplementary File 6. Subject HOKD0485FX relative fitness estimate, co-occurring mutations and frequency of occurrence for each reconstructed haplotype.**

| **Region** | **Time** | **Viral Load*** | **Frequency** | **Relative Fitness** | **_1395_HSKKKCDEL_1403_**  **Mutations** | | **Co-occurring Mutations**** | | |
| --- | --- | --- | --- | --- | --- | --- | --- | --- | --- |
| NS3 | 30DPI | 733849 | 13.10% | 1.0000 |  |  |  |  |  |
|  |  |  | 13.00% | 0.1509 |  |  |  | D1605G |  |
|  |  |  | 11.70% | 0.1509 |  |  |  | C1518F |  |
|  |  |  | 9.80% | 0.1509 |  |  |  | D1605G |  |
|  |  |  | 5.10% | 0.1509 |  |  |  | D1605G |  |
|  |  |  | 5.10% | 1.0000 |  |  |  |  |  |
|  |  |  | 4.90% | 0.1509 |  |  |  | C1518F |  |
|  |  |  | 4.50% | 1.0000 |  |  |  |  |  |
|  |  |  | 4.00% | 1.0000 |  |  |  |  |  |
|  |  |  | 3.60% | 1.0000 |  |  |  |  |  |
|  |  |  | 2.30% | 0.0336 |  |  |  | A1230T | A1302T |
|  |  |  | 2.20% | 0.0144 |  |  |  | S1536P |  |
|  |  |  | 2.10% | 0.1511 |  |  |  | T1286P |  |
|  |  |  | 1.60% | 0.1199 |  |  |  |  |  |
|  |  |  | 1.60% | 0.0350 |  |  |  | V1198A | I1314M |
|  |  |  | 1.60% | 0.0228 |  |  |  | V1198A |  |
|  |  |  | 1.40% | 0.1509 |  |  |  | P1241S | A1302T |
|  |  |  | 1.20% | 0.1509 |  |  |  | G1233D |  |
|  | 72DPI | 175219 | 66.90% | 0.2373 | K1398R |  |  |  |  |
|  |  |  | 7.50% | 0.2373 | K1398R |  |  |  |  |
|  |  |  | 7.20% | 0.2373 | K1398R |  |  |  |  |
|  |  |  | 6.40% | 0.0358 | K1398R |  |  | G1307E |  |
|  |  |  | 3.10% | 0.0364 | K1398R |  |  | S1215F |  |
|  |  |  | 2.60% | 0.2373 | K1398R |  |  |  |  |
|  |  |  | 2.40% | 0.0359 | K1398R |  | Y1644C |  |  |
|  |  |  | 2.20% | 0.2373 | K1398R |  |  |  |  |
|  |  |  | 1.60% | 0.0245 | K1398R |  |  | T1459I |  |
|  | 79DPI | 44452 | 36.70% | 0.2373 | K1398R |  |  |  |  |
|  |  |  | 23.80% | 0.2373 | K1398R |  |  |  |  |
|  |  |  | 23.80% | 0.2373 | K1398R |  |  |  |  |
|  |  |  | 15.70% | 0.2373 | K1398R |  |  |  |  |
|  | 93DPI | 407392 | 41.30% | 0.3420 | K1398R |  | V1641I |  |  |
|  |  |  | 27.30% | 0.0324 | K1398R |  | V1641I | A1113T |  |
|  |  |  | 11.20% | 0.0516 | K1398R |  | V1641I | A1085V |  |
|  |  |  | 7.40% | 0.0049 | K1398R |  | V1641I | A1085V | A1113T |
|  |  |  | 3.40% | 0.3420 | K1398R |  | V1641I |  |  |
|  |  |  | 2.30% | 0.0324 | K1398R |  | V1641I | A1113T |  |
|  |  |  | 2.10% | 0.2373 | K1398R |  |  |  |  |
|  |  |  | 1.70% | 0.1189 | K1398R |  | V1641I | R1496G |  |
|  |  |  | 1.60% | 0.3420 | K1398R |  | V1641I |  |  |
|  |  |  | 1.50% | 0.0223 | K1398R |  | V1641I | A1113T |  |
|  | 107DPI | 24969 | 100.00% | 0.0039 | D1401N |  | V1641I |  |  |
|  | 121DPI | 77723 | 19.00% | 0.0034 | K1398R | K1397R | V1641A |  |  |
|  |  |  | 11.80% | 0.0034 | K1398R | K1397R | V1641A |  |  |
|  |  |  | 10.80% | 0.0034 | K1398R | K1397R | V1641A |  |  |
|  |  |  | 7.50% | 0.0034 | K1398R | K1397R | V1641A |  |  |
|  |  |  | 5.90% | 0.0034 | K1398R | K1397R |  |  |  |
|  |  |  | 4.30% | 0.0285 | K1398R | K1397R | V1641A |  |  |
|  |  |  | 4.30% | 0.0034 | K1398R | K1397R | V1641A |  |  |
|  |  |  | 4.00% | 0.0034 | K1398R | K1397R | V1641A |  |  |
|  |  |  | 3.10% | 0.0295 | K1398R |  | V1641A |  |  |
|  |  |  | 2.90% | 0.0148 | K1398R | K1397R | V1641A |  |  |
|  |  |  | 2.60% | 0.0005 | K1398R | K1397R | V1641A | I1285V |  |
|  |  |  | 2.50% | 0.0285 | K1398R | K1397R |  |  |  |
|  |  |  | 2.40% | 0.0285 | K1398R | K1397R |  |  |  |
|  |  |  | 2.30% | 0.0073 | K1398R | K1397R | V1641T |  |  |
|  |  |  | 2.00% | 0.0034 | K1398R | K1397R | V1641A |  |  |
|  |  |  | 2.00% | 0.0034 | K1398R | K1397R | V1641A |  |  |
|  |  |  | 2.00% | 0.0295 |  | K1397R | V1641A |  |  |
|  |  |  | 1.90% | 0.0295 |  | K1397R | V1641A |  |  |
|  |  |  | 1.90% | 0.0005 | K1398R | K1397R | V1641A | A1405V |  |
|  |  |  | 1.80% | 0.0148 | K1398R |  | V1641A |  |  |
|  |  |  | 1.80% | 0.0005 | K1398R | K1397R | V1641A | I1285V |  |
|  |  |  | 1.70% | 0.0148 | K1398R |  | V1641A |  |  |
|  |  |  | 1.70% | 0.0073 | K1398R | K1397R | V1641T |  |  |
|  | 149DPI | 254245 | 97.10% | 0.0031 | K1398R | K1397R | M1646T |  |  |
|  |  |  | 1.80% | 0.0005 | K1398R | K1397R | M1646T | A1597T |  |
|  |  |  | 1.10% | 0.0005 | K1398R | K1397R | M1646T | G1489D |  |
|  | 233DPI | 350658 | 62.00% | 0.0002 | K1398R | K1397R | M1646T |  |  |
|  |  |  | 12.00% | 0.0002 | K1398R | K1397R | M1646T |  |  |
|  |  |  | 3.60% | 0.0002 | K1398R | K1397R | M1646T | A1647T |  |
|  |  |  | 3.50% | 0.0002 | K1398R | K1397R | M1646T |  |  |
|  |  |  | 3.20% | 0.0000 | K1398R | K1397R | M1646T | K1162R |  |
|  |  |  | 3.10% | 0.0002 | K1398R | K1397R | M1646T |  |  |
|  |  |  | 2.70% | 0.0015 | K1398R | K1397R | M1646T | V1329I |  |
|  |  |  | 2.70% | 0.0003 | K1398R | K1397R | M1646T | V1641I |  |
|  |  |  | 2.60% | 0.0002 | K1398R | K1397R | M1646T |  |  |
|  |  |  | 2.50% | 0.0002 | K1398R | K1397R | M1646T |  |  |
|  |  |  | 2.20% | 0.0002 | K1398R | K1397R | M1646T | K1088R |  |
| *Viral Load measured in IU/ML. | | | | | | | | |  |
| **Only non-synonymous mutations are shown. | | | | | | | | |  |
